# Supplementary material for: Effort-Reward Imbalance, Over-Commitment and Depressive Episodes at Work: Evidence from the ELSA-Brasil Cohort Study
Source: Int J Environ Res Public Health. 2019 Aug 21;16(17):3025. doi: 10.3390/ijerph16173025 (PMC6747529; doi:10.3390/ijerph16173025)
Supplement: Supplementary file 1 [file ijerph-16-03025-s001.pdf]

Supplement Table

Table 1. - Association between 'reward' subscales and depressive episodes including prevalence ratio (PR), and 95% confidence interval.

Scales	Prev. (%)	Model 1		Model 2	
		PR	IC95%	PR	IC95%
**Esteem**					
Quartile 1	8.80	3.27	2.43-4.13	3.08	2.36-4.03
Quartile 2	5.15	1.85	1.39-2.48	1.79	1.34-2.41
Quartile 3	3.29	1.18	0.87-1.60	1.23	0.91-1.66
Quartile 4	2.52	-	-		-
**Job Promotion**					
Quartile 1	8.59	2.66	2.06-3.42	2.51	1.94-3.25
Quartile 2	5.75	1.58	1.36-2.32	1.70	1.30-2.23
Quartile 3	3.65	1.12	0.85-1.48	1.02	0.86-1.49
Quartile 4	3.23	-	-	1.00	-
**Job Insecurity***					
(Undesirable change)					
Strongly agree	8.27	3.25	2.39-4.41	2.99	2.19-4.10
Agree	4.89	1.92	1.42-2.59	1.81	1.33-2.47
Disagree	3.36	1.32	0.91-1.91	1.32	0.91-1.92
Strongly disagree	2.54	-	-	1.00	-

* In this ERI subscale only one question of the two original "job insecurity" scale components was included: "I have experienced or I expect to experience an undesirable change in my work situation".
Model 1. Crude model; Model 2. Adjusted by age, gender, marital status, race/skin color, education.

Table 2. - Association between 'effort'-'reward' imbalance and depressive episodes including prevalence ratio (PR) and respective 95% confidence intervals according to gender. ELSA-Brasil, 2012-2014.

	Men		Women	
Scales	PR*	IC95%	PR*	IC95%
**Effort**				
Quartile 1	1.00	--	1.00	--
Quartile 2	1.47	0.90-2.39	1.13	0.83-1.55
Quartile 3	1.71	1.10-2.66	1.23	0.93-1.63
Quartile 4	2.16	1.33-3.50	1.74	1.30-2.32
**Reward**				
Quartile 1	4.20	2.32-7.59	3.19	2.25-4.50
Quartile 2	2.07	1.07-4.02	1.49	1.00-2.23
Quartile 3	1.67	0.85-3.26	1.45	0.96-2.18
Quartile 4	1.00	--	1.00	--
**Over-commitment**				
Quartile 1	1.00	--	1.00	--
Quartile 2	1.03	0.54-1.95	1.21	0.86-1.71
Quartile 3	3.25	1.89-5.59	1.77	1.24-2.51
Quartile 4	4.27	2.81-7.74	3.27	2.42-4.42
**ER Ratio**				
Quartile 1	1.00	--	1.00	--
Quartile 2	1.55	0.89-2.72	0.80	0.56-1.16
Quartile 3	1.75	1.00-3.06	1.00	0.71-1.41
Quartile 4	3.34	2.02-5.55	2.19	1.64-2.91

* Model adjusted by age, marital status, race/skin color, education.
